# Tamoxifen-related endocrine symptoms in Chinese patients with breast cancer: Study protocol clinical trial (SPIRIT Compliant)

**DOI:** 10.1097/MD.0000000000019083

**Published:** 2020-02-21

**Authors:** Carmen Wing Han Chan, Christine Miaskowski, Alexandra McCarthy, Mary Miu Yee Waye, Winnie Yeo, Winnie Kwok Wai So, Kai Chow Choi, Stephen Kwok Wing Tsui, Judy Yuet Wa Chan

**Affiliations:** a The Nethersole School of Nursing, The Chinese University of Hong Kong, Hong Kong; b School of Nursing, University of California, San Francisco, CA; c School of Nursing, Midwifery and Social Work, University of Queensland, Queensland, Australia; d The Croucher Laboratory for Human Genomics, The Chinese University of Hong Kong; e Department of Clinical Oncology; f School of Biomedical Sciences, The Chinese University of Hong Kong, Hong Kong.

**Keywords:** breast cancer, drug adherence, endocrine symptoms, single nucleotide polymorphism, tamoxifen

## Abstract

**Background:**

Breast cancer is the most prevalent cancer in females and disease recurrence remains a significant problem. To prevent recurrence, tamoxifen is prescribed for at least 5 years. However, among patients who receive tamoxifen, individual responses are highly variable. These responses are affected by the type, frequency, and severity of endocrine symptoms, as well as adherence rates. Polymorphisms in genes involved in the metabolism of tamoxifen (ie, CYP3A4, CYP2D6) may influence responses to tamoxifen. In this study, the inter-relationships among endocrine symptoms, drug adherence, and genetic polymorphisms in Chinese breast cancer patients receiving tamoxifen therapy will be examined. We hypothesize that patients with more severe endocrine symptoms will be less likely to adhere to tamoxifen treatment. In addition, we hypothesize that a relationship will exist between the severity of tamoxifen-induced symptoms and allelic variations in tamoxifen metabolism-related genes. Although many association studies have determined that select genotypes influence the efficacy of tamoxifen, very few studies have investigated for associations between tamoxifen-induced endocrine symptoms and these polymorphisms.

**Objectives:**

The aim of this study was to characterize genetic polymorphisms in tamoxifen metabolism-associated genes in Chinese women with breast cancer and to explore the inter-relationships between genetic polymorphisms, endocrine symptoms, and adherence to tamoxifen.

**Method:**

We will conduct a prospective cohort study that follows 200 Chinese women over 18 months and assess treatment-related symptoms and genetic variations. Endocrine symptoms and drug adherence will be determined through interview-administered standardized questionnaires. Polymorphisms in drug metabolism genes will be determined using real-time polymerase chain reaction based genotyping method. Data will be analyzed to determine associations between allelic variations, endocrine symptoms, and adherence.

**Discussion:**

The proposed study will evaluate for polymorphisms in gene(s) that are associated with tamoxifen-related endocrine symptoms and adherence with tamoxifen. We will explore the relationships between genotypes, endocrine symptoms, and drug adherence in Chinese breast cancer patients. Findings from this study may assist clinicians to identify patients at higher risk for a worse symptom experience and lower adherence rates and enable them to initiate appropriate interventions. In the long term, the findings from this study may be used to develop and test tailored symptom management interventions for these patients.

## Abstract 2

Supplemental Digital Content is available in the text

## 1 Introduction

Breast cancer is the most common female cancer worldwide. In 2018, the Global Cancer Observatory estimated that slightly >2 million new cases of breast cancer were diagnosed in women worldwide and approximately 600,000 patients died from the disease.^[1]^ In Hong Kong, the incidence of breast cancer increased from 1152 in 1993 to 4108 in 2016. On average, about 9 women are diagnosed with breast cancer every day in Hong Kong. These women's median age is 56 years compared to 61 years in the United States and 62 years in Australia. The rising incidence of breast cancer in Hong Kong, particularly in younger women, is attributed to the increased Westernization of Chinese women, particularly in terms of adverse dietary and other lifestyle practices.^[2]^

In estrogen receptor positive (ER+) early-stage breast cancer, 5 years of adjuvant endocrine therapy substantially reduce the risks of locoregional and distant recurrence, contralateral breast cancer, and death.^[3–5]^ Tamoxifen, a selective estrogen receptor (ER) modulator (SERM), is the most frequently used antiestrogen adjuvant treatment for ER+ pre-menopausal women. In trials that compared 5 years of tamoxifen therapy to no endocrine therapy in patients with ER+ breast cancer, the recurrence rate in the tamoxifen group was approximately 50% lower during the first 5 years and approximately 30% lower during the subsequent 5 years. In addition, the mortality rate was approximately 30% lower in the tamoxifen group during the first 15 years.^[4]^ However, tamoxifen had little or no effect on breast cancer recurrence or mortality in ER-negative disease.

To maximize its effectiveness, tamoxifen should be taken at the recommended dose for 5 years. However, effectiveness is predicated on 2 inter-related factors. The first is individual drug metabolism, which affects the response of the cancer to treatment.^[6]^ The second is drug adherence, which is markedly influenced by the type and severity of tamoxifen-related symptoms.^[7]^ Previous studies mainly focused on the impact of genotypes on tamoxifen metabolism and efficacy.^[8,9]^ In view of the importance of drug adherence, a need exists to identify biomarkers that are associated with tamoxifen-related symptoms which affect patients’ level of adherence.

Tamoxifen's endocrine symptom profile can influence patients’ perceptions of the drug's benefits or harms. If symptoms are severe, patients may not adhere to tamoxifen. Common endocrine symptoms associated with tamoxifen include vasomotor symptoms (eg, hot flashes, cold sweats, night sweats), neuropsychiatric symptoms (eg, dizziness, headaches, mood swings, anxiety, depression, insomnia), musculoskeletal symptoms (eg, muscle and joint pain), and sexual dysfunction. These symptoms can be disabling. In 1 study, the most common symptom was hot flashes (64%), with 20% of women reporting severe hot flashes.^[10]^ However, because the type and severity of symptoms are highly variable among patients, clinicians are not able to predict which patients will experience a higher symptom burden.

Adherence rates are usually reported as the percentage of the prescribed dose of the medication actually taken by the patient over a specified period of time.^[11]^ Given the large amount of interindividual variability in tamoxifen-related endocrine symptoms and the need to take the drug daily for 5 years, different adherence rates were reported for different age groups and treatment durations. In 1 study of 5707 patients who took tamoxifen for 5 years, the incidence of nonadherence with tamoxifen in women <35 years of age at 1 year was 11%, increasing to approximately 17%, 23%, and 25% at 2, 3, and 4 years after random assignment. For those ≥35 years of age, nonadherence rates were 9%, 14%, 18%, and 21%, respectively.^[12]^

This evaluation of adherence is important because nonadherence results in higher mortality. For example, estimated survival at 10 years was 80.7% for women who continued hormonal therapy versus 73.6% for those who discontinued it (*P* < .001). Of those who continued, survival at 10 years was 81.7% and 77.8% in women who adhered and nonadhered, respectively (*P* < .001). Adjusting for demographic and clinical characteristics, both early discontinuation (hazard ratio [HR] 1.26, 95% confidence interval [CI] 1.09–1.46) and nonadherence (HR 1.49, 95% CI 1.23–1.81), among those who continued, were independent predictors of mortality.^[13,14]^ In addition, poor drug adherence may lead to disease progression, complications, and additional medical costs.^[15]^ Given the significant survival benefits associated with tamoxifen, understanding the extent of adherence to endocrine therapies and identifying genetic and modifiable factors (eg, forgetting to take medication while travelling) that influence adherence warrant investigation.

Patients with very similar clinical characteristics and prognostic factors at diagnosis, who receive the same tamoxifen regimen, vary substantially in their treatment responses and in the clinical course of their disease.^[16]^ These responses can be partially explained by polymorphisms in three tamoxifen metabolism-related genes: cytochrome P450 enzymes, UDP-glucuronosyltransferase and sulfotransferase, and ATP-binding cassette transporters.

Tamoxifen is metabolized into its more active forms by various cytochrome P450 enzymes (eg, CYP2D6, CYP3A4, CYP3A5, CYP2C9, and CYP2C19).^[17,18]^ The effects of CYP polymorphisms on tamoxifen pharmacokinetics were well studied in Asian countries.^[19,20]^ In addition, the UDP-glucuronosyltransferase (primarily UGT1A8, UGT1A10, UGT2B7 and UGT2B15) and sulfotransferase (primarily SULT1A1) catalyze the conversion of tamoxifen metabolites into excretable forms. Sulfotransferase enzymes catalyze the transfer of a sulfonyl group to nucleophilic groups increasing their solubility and facilitating their excretion.^[21,22]^ Finally, 3 members of the ATP-binding cassette (ABC) transporters, namely, ABCB1 (P-gp/MDR1), ABCC1 (BRCP), and ABCC2 (MRP2), are key players in the drug resistance phenotype. ABCB1 is expressed in 28% to 63% of breast cancers, depending on the methodology applied. Polymorphisms in the genes that encode for these transporter proteins can increase their expression and reduced the effectiveness of tamoxifen.

All of the enzymes and transporters in tamoxifen's metabolic pathway are polymorphic. Thus, inter-individual differences in tamoxifen metabolism contribute to variations in the concentration of metabolites in the serum and the drug's effectiveness.

As part of the proposed study, we hypothesize that the drug's metabolic rate may influence the occurrence and severity of tamoxifen-induced endocrine symptom. Only two studies were identified that support this hypothesis. In the first study of 297 patients with breast cancer who were genotyped for polymorphisms in CYP2D6,^[23]^ compared to poor metabolizers (*P* = .038) and extensive metabolizers (*P* = .011), intermediate metabolizers had significantly higher hot flash severity score after 4 months of tamoxifen treatment. In the second study of 258 patients with breast cancer who were genotyped for polymorphism in CYP2D6 and ABCB1,^[24]^ no association were found between any of the polymorphisms and associated symptoms. A major limitation of this second study is that a valid and reliable assessment tool was not used to collect data on tamoxifen-induced endocrine symptoms. Given the positive results from the first study,^[23]^ additional research is warranted to confirm or refute our hypothesis.

Based on the results of previous studies,^[17,18,23–25]^ potential alleles will be selected to test their relationship with symptoms in breast cancer patients. In this study, we aim to characterize polymorphisms in tamoxifen metabolism associated genes and to evaluate for associations among genetic polymorphisms, endocrine symptoms, and adherence to tamoxifen in Chinese patients with breast cancer.

## 2 Methods

### 2.1 Study design

This prospective cohort study will recruit patients from the Department of Clinical Oncology in Prince of Wales Hospital in Hong Kong. Patients will be enrolled within 30 days of starting tamoxifen. Patients will complete self-report questionnaires on endocrine symptoms and a saliva sample will be collected at enrolment. At 3, 6, 9, 12, 15, and 18 months after enrolment, data on symptoms and drug adherence will be collected through phone interviews using the same measures. Data collection will end at 18 months because symptoms of tamoxifen therapy appear early and are worst during the first 12 months after the initiation of treatment.^[26]^ Patients’ saliva will be collected during enrolment for evaluation of single-nucleotide polymorphism (SNPs) in genes associated with drug metabolism enzymes and transporters.

### 2.2 Patient recruitment

A total of 200 Chinese women who received primary therapy for breast cancer and commence tamoxifen within the past month will be recruited for this study. The inclusion criteria for the patients are: Chinese women with histologically confirmed estrogen receptor-positive, stage I–III, primary invasive breast cancer treated with definitive surgery and/or chemotherapy and started on tamoxifen (20 mg daily) within the past month. We will exclude patients with other cancers within the last 5 years. In addition, patients who are pregnant or are planning to become pregnant, lactating, treated with investigational drugs within the 4 weeks before enrolment, as well as those who are not able to provide written informed consent will be excluded. The participating hospitals have 600 new cases of breast cancer per year and half of these patients will be prescribed tamoxifen. It is anticipated that 100 to 150 patients will be recruited per year based on our experience with the pilot study.

### 2.3 Ethical considerations

This study was registered on the ISRCTN Registry with reference number ISRCTN10773849. The study protocol was approved by the Joint Chinese University of Hong Kong—New Territories East Cluster Clinical Research Ethics Committee with reference number 2016.554. Any modifications to the protocol that may have an impact on the conduct of the study will be reported to the committee. Potential patients will be given detailed information about the study and their rights to participate (Supplemental Digital Content (Appendix 1)). Written informed consent will be obtained before data collection (Supplemental Digital Content (Appendix 2)).

### 2.4 Sample size estimation

The sample size was calculated to provide adequate power to examine associations between genetic polymorphisms and severity of endocrine symptoms associated with tamoxifen treatment, as well as adherence with tamoxifen treatment. In general, a larger sample size for an association analysis allows for the detection of a smaller effect size at a specific statistical power and level of significance. However, a small effect size may not be of clinical importance. In consideration of both clinical relevance and statistical power, we aim to detect a reasonable effect size of *R*^2^ = 0.05 for a genetic predictor of severity of endocrine symptoms of tamoxifen treatment as well as for an association between the severity of endocrine symptoms and level of adherence with tamoxifen treatment.

Using PASS 13 (NCSS, Kaysville, UT), it is estimated that a sample size of 152 patients would give our study 80% power at a 5% level of significance to detect an effect size of *R*^2^ = 0.05 (ie, if a specific polymorphism explains at least 5% of the variance in the severity of symptoms of tamoxifen treatment or if the severity of symptoms explains 5% of variance in adherence to the treatment, they will be detectable with 80% power at 5% significance level).

Since our follow-up measurements are based on brief telephone interviews, we anticipate that the nonresponse rate will be relatively low. In fact, all of the patients completed the 3-month follow-up in our ongoing pilot study. Furthermore, on the basis of a 1-year crude death rate of breast cancer patients in Hong Kong (4.4%) (WHO, http://survcan.iarc.fr/index.php) and assuming an average non-response rate for our follow-up questionnaires of <3% for every 3 months, we anticipate that the overall attrition rate in our 18-month prospective cohort study will be 24%. Therefore, we will recruit 200 eligible patients into the study.

### 2.5 Clinical assessments

Three kinds of clinical data will be collected from the patients.

#### 2.5.1 Symptoms

Endocrine symptoms will be assessed using the Greene Climacteric Scale (GCS) and the Functional Assessment of Cancer Therapy-Endocrine subscale (FACT–ES) questionnaire (version 4) from the FACIT system. The GCS is a 21-item self-administered instrument that contains 4 domains (ie, psychological, somatic, vasomotor, sexual). Each item is rated on a 3-point scale (1 = a little to 3 = extremely), indicating the severity of the symptoms experienced. The mean score for each domain will be calculated. The GCS has good internal consistency and test–retest reliability.^[27]^ Psychometric properties of the Chinese version were reported previously.^[28]^

The FACT-ES is a 19-item questionnaire that measures menstrual symptoms, such as vaginal dryness and spotting. These menstrual symptoms are not included in the GCS. The internal consistency and test–retest reliability of the Chinese version of the FACT-ES is published.^[29]^ Patients will be interviewed using the GCS and FACT-ES at enrolment and again at 3, 6, 9, 12, 15, and 18 months.

#### 2.5.2 Drug adherence

The Modified Medication Adherence Report Scale (MMARS) will assess the patients’ level of adherence with tamoxifen. The Medication Possession Ratio (MRP) will be used to cross-validate this self-report measure. MARS assesses patients’ attitudes toward medication, perceived benefits from medications, and insights into their illness. The modified MARS (MMARS) contains 6 items selected from the 22-item MARS that pertains to patients’ level of adherence with medication behaviors and attitudes. The Cantonese version of this scale will be used in this study.^[30]^

The MPR^[31]^ is calculated as the sum of the day's supply of all relevant medications during a defined period of time divided by the number of days elapsed during that period of time. This ratio is used frequently to evaluate drug adherence. Given the variability in the severity of tamoxifen-related endocrine symptoms and the need to take the drug daily for a long period of time, MPR rates will be defined as good if >72% of the tamoxifen is taken, normal if 41% to 72% is taken, and poor if <41% is taken.^[14,32–34]^ Patients will be asked to complete a medication diary on a daily basis to record the intake of tamoxifen, supplements, Chinese herbal medicines, and other medications, and to complete the drug refill form. This information will be collected every 3 months by a research assistant.

#### 2.5.3 Demographic and clinical characteristics

Demographic and clinical characteristics will be collected at enrolment by self-report. This information includes: education and socioeconomic status, height and weight, menopausal status, smoking, alcohol use, parity, level of physical activity, medications, comorbidities, self-medications (eg, home remedies, over the counter remedies, dietary supplements, Chinese medicine), stage of breast cancer, tested for BRCA1/2, family history of breast cancer, and history of oophorectomy. At 12 and 18 months, information on breast cancer recurrence will be collected during the phone interview.

### 2.6 Genomic assessment

At enrolment, saliva samples will be collected from patients and stored in a −80°C freezer. Total genomic DNA will be extracted from the saliva using QIAamp DNA Mini Kit (Qiagen, USA) as per supplier's instructions. Genotype analyses will be carried out with ready-to-order TaqMan assays from Thermo Fisher. All patients will be tested for allelic variations in 2 cytochrome P450 enzyme genes (ie, UDP glucuronosyl transferase and sulfotransferase) and 14 drug transporter genes (ie, CYP3A4, CYP3A5, CYP2C9, CYP2C19, CYP2D6, CYP1A2, CYP2B6, UGT1A4, UGT2B7, UGT1A8, UGT2B15, ABCC1, ABCB1, ABCC2). These SNPs were selected because they were reported to be involved in tamoxifen metabolism, which may affect the occurrence and severity of endocrine symptoms.^[20,22]^

Based on the available genotype data, CYP2D6 alleles (as an example) will be assigned a value that reflects the expected activity of the CYP2D6 enzyme for which they code. For example, fully functional CYP2D6 alleles (eg, ∗1, ∗2, ∗33, ∗35) will be assigned a score of 1. CYP2D6 alleles associated with reduced enzyme activity (eg, ∗9,∗10, ∗17, ∗29, ∗37, ∗41, ∗45, ∗46) will be scored as 0.5. The null CYP2D6 alleles (eg, ∗3-∗8, ∗11-∗16, ∗18-∗20, ∗38, ∗40, ∗42, ∗44, ∗56) and their duplications will receive a value of 0. Duplications of fully active alleles (eg, ∗1×N, ∗2 × N, ∗35 × N) will be assigned a value +1, rendering a score of 2 for each of these duplicated variants. Duplicated reduced activity alleles (eg, ∗41 × N and ∗45 × N) will be assigned a value of +0.5, yielding a score of 1.^[35]^

### 2.7 Data analysis

Data will be summarized using appropriate descriptive statistics. Continuous data will be assessed for skewness and kurtosis and normal probability. Appropriate transformations will be made for skewed variables before entering them into the statistical analysis.

Since an episode of drug adherence might not reflect an overall adherence rate of a medication for an extended duration, a summary measure based on the area under the curve (AUC) of repeated measures of tamoxifen treatment adherence assessed using MMARS over 18 months (ie, specifically at months 3, 6, 9, 12, 15, 18) will be used to quantify overall adherence with tamoxifen treatment. Likewise, the AUC for each of the items on the GCS and the FACT-ES that are measured at enrolment, 3, 6, 9, 12, 15, 18 months will be computed first to obtain an overall measure of the severity of each symptom.

Since some highly correlated items may exist in the GCS and FACT-ES, a principal component factor analysis will be conducted on the AUCs of the GCS and FACT-ES items to identify uncorrelated principal components. These principal components can be regarded as the latent factors that give rise to the reported symptoms. Since no previous evidence exists regarding which symptom has a larger effect on adherence with tamoxifen, equal weighting will be given to each principal component. Then, the standardized factor scores computed using the Anderson-Rubin method of the principal components identified will be summed to quantify the overall severity of the symptoms associated with tamoxifen treatment.

The genetic factors will be examined as individual SNPs and haplotypes of SNPs from different candidate loci, as appropriate. The distribution of genotypes in candidate SNPs will be tested for Hardy–Weinberg equilibrium (HWE) using Fisher exact test. SNPs in conformation with HWE will be used in regressions analyses to determine their relative contribution to the overall severity of the symptoms associated with tamoxifen treatment with adjustment for potential confounders, including age, menopausal status, stage of cancer at diagnosis, and treatment received. The linkage disequilibrium between SNPs in candidate loci will be analyzed using Haploview (Daly Lab, Cambridge, MA) and haplotypes will be created. The relationships between these haplotypes and overall severity of the symptoms of tamoxifen will be analyzed by the regression-based approach of Zaykin et al^[36]^ after adjusting for potential confounders with the use of a permutation test. Statistical significance will be evaluated using permutations with 10,000 replications. Inflated overall type I error rate will be controlled using the approach of Benjamini and Hochberg.^[37]^ Furthermore, multivariable regression analysis will examine the associations between overall adherence with tamoxifen treatment and overall severity of the symptoms of tamoxifen treatment with adjustment for the above potential confounders. All statistical analyses unless otherwise specified will be performed using SAS release 9.4 (SAS Institute, Cary, NC). All statistical tests will be 2-sided and a *P* value <.05 will be considered statistically significant.

## 3 Discussion

Adjuvant tamoxifen, an essential treatment for ER+ breast cancer, has been used for >3 decades to reduce the risk of breast cancer recurrence and mortality.^[38]^ To ensure optimal benefits, patients must take tamoxifen for at least 5 years.^[39]^ Despite the acknowledged benefits of both reduced recurrence and increased survival rates, adherence to tamoxifen is less than ideal. Approximately 1 in 5 patients who are prescribed tamoxifen do not achieve the optimal adherence threshold of ≥80% during the first year of treatment, with a subsequent 7% to 10% discontinuation rate per year. By the fourth or fifth year of treatment, the adherence rate is as low as 50%.^[40]^ The most significant factor that contributes to nonadherence is the tamoxifen-related endocrine symptom profile. Symptoms include sudden, severe, and often permanent vasomotor symptoms and related insomnia, somatic symptoms, depression, and sexual dysfunction.^[41]^

Adjuvant endocrine therapy is associated with many adverse effects that can significantly decrease patients’ quality of life and lead to substantial economic loss because of medical expenses and inability of patients to function well in their existing roles in family and in society.^[42]^ Untreated side effects may lead to early discontinuation of treatment and poor adherence, which could compromise overall survival. With >3 million breast cancer survivors and longer survival times with current treatment alternatives, management of survivors’ health issues will increasingly fall to primary care clinicians. The most effective management is a multifactorial approach that addresses patients’ symptoms, health promotion, and referrals.^[42]^

The optimal management strategy for tamoxifen is one that considers the balance between the drug's potential benefits and adverse effects. The way that tamoxifen is metabolized and its potential toxicities are partially influenced by each patient's genetic makeup.^[43]^ The aim of this study is to determine whether, and how, symptoms of tamoxifen correlate with drug adherence and with polymorphisms in genes that regulate the metabolism of tamoxifen in Chinese breast cancer patients. We hypothesize that patients with more severe endocrine symptoms (ie, psychological, somatic, vasomotor, sexual) are less likely to adhere to tamoxifen treatment. In addition, we hypothesize that a relationship exists between the severity of tamoxifen-induced symptoms and allelic variations in tamoxifen metabolism-related genes. To date, studies have focused primarily on the associations between genetic polymorphisms and tamoxifen efficacy^[44,45]^ and not on the association between these factors and tamoxifen-induced symptoms and subsequent adherence. Adherence could be improved by understanding the factors that are associated with patients who are less likely to adhere and any modifiable factors (eg, barriers and facilitators) to their adherence.^[46]^ This increased understanding could contribute to the development of individualized symptom management interventions for patients who are prescribed tamoxifen, ultimately leading to greater adherence and reduced risks of breast cancer recurrence and death.

This study is significant in that it will provide preliminary evidence on the factors associated with tamoxifen adherence in Chinese breast cancer patients. The results of this study may lead to larger descriptive and interventional studies that will evaluate different approaches to increase tamoxifen adherence rates and its clinical efficacy.

It is increasingly recognized that people are prescribed medications that will not help them and could even harm them. The increased recognition that clinicians must account for individual variability in therapeutic effects and symptoms is driving the recent momentum in ’precision’ health care.^[47]^ This more personalized approach is informed by a different type of clinical trial that focuses on individual, not average, responses to therapy and which probes the specific factors that influence patients’ responses to therapy, including genetic and behavioral, among others.^[48]^ In this study, we will consider the relationships between three critical factors (ie, endocrine symptoms of tamoxifen, drug adherence, genetic polymorphisms) that may influence the therapeutic outcomes of adjuvant tamoxifen (eg, disease recurrence, mortality). In the long term, this project is a stepping stone in the further development of personalized adjuvant tamoxifen and tailored symptom management interventions for breast cancer patients based on their individual genomic profile.

This study started in November 2016 and is on-going status at the present time.

## Author contributions

Carmen Chan, Christine Miaskowski, Alexandra McCarthy and Winnie So are responsible for study design.

Mary Waye and Stephen Tsui provide suggestions on genetic polymorphism analysis.

Winnie Yeo provides suggestions on recruitment of patients.

KC Choi is responsible for data analysis.

Judy Chan is responsible for phone interview and SNP analysis.

All of the authors contributed to revisions of the manuscript and its final approval.

## Supplementary Material

Supplemental Digital Content

## Supplementary Material

Supplemental Digital Content
