# Supplementary material for: Tamoxifen-related endocrine symptoms in Chinese patients with breast cancer: Study protocol clinical trial (SPIRIT Compliant)
Source: Medicine (Baltimore). 2020 Feb 21;99(8):e19083. doi: 10.1097/MD.0000000000019083 (PMC7034730; doi:10.1097/MD.0000000000019083)
Supplement: Supplemental Digital Content [file medi-99-e19083-s001.docx]

**Appendix 1**

Subject Code: ________

The Chinese University of Hong Kong

Faculty of Medicine

The Nethersole School of Nursing

*Consent form*

**Study title**:

Advancing the science of personalized health care: A prospective study to uncover the role of predictive biomarkers in tamoxifen-related endocrine symptoms and drug adherence based on genetic polymorphisms in breast cancer patients in Hong Kong

I confirm that I have read and be informed of the purpose of the study, the procedure that I will undergo, the risk and benefit that I may experience. I have had opportunities to ask questions which have been explained to my satisfaction. I understand that participation is entirely voluntary. I have the right to decline the participation at any time without providing any reasons. I also understand that all the information I give will be used only in this research, kept strictly confidential and that I will not be in any way identifiable. I also understand that sections of any of my medical notes may be looked at by responsible individuals from regulatory authorities (including Joint CUHK-NTEC CREC) where it is relevant to my taking part in research for ethics review purpose. I give permission for these individuals to have access to my records. I have read this consent form and I understand this consent form. Therefore, I agree to give my consent to participate in this study.

If I have any questions of the subject rights, I may contact the Joint CUHK-NTEC Clinical Research Ethics Committee at 3505 3935 for clarification

___________________ _____________________ _________________________

Signature of participant Signature of witness Signature of investigator

___________________ _____________________ _________________________

Name of participant Name of witness Name of investigator

___________________ _____________________ _________________________

Date Date Date
