# Supplementary material for: Tamoxifen-related endocrine symptoms in Chinese patients with breast cancer: Study protocol clinical trial (SPIRIT Compliant)
Source: Medicine (Baltimore). 2020 Feb 21;99(8):e19083. doi: 10.1097/MD.0000000000019083 (PMC7034730; doi:10.1097/MD.0000000000019083)
Supplement: Supplemental Digital Content [file medi-99-e19083-s002.docx]

**Appendix 2**

The Chinese University of Hong Kong

Faculty of Medicine

The Nethersole School of Nursing

Subject Code:____________

*Information Sheet*

**Study title**

Advancing the science of personalized health care: A prospective study to uncover the role of predictive biomarkers in tamoxifen-related endocrine symptoms and drug adherence based on genetic polymorphisms in breast cancer patients in Hong Kong

**Principal Investigator**

Prof Carmen WH Chan

Professor, the Nethersole School of Nursing, Faculty of Medicine, the Chinese University of Hong Kong

**The Study Background**

Breast cancer is the most prevalent cancer in females in Hong Kong, and worldwide. After primary therapy such as surgery and chemotherapy, disease recurrence remains a significant concern. Nowadays, a common practice to prevent recurrence is to have women on adjuvant tamoxifen, a hormonal therapy, for five years. The relapse rate at 5 years for patients who adhere to adjuvant tamoxifen is significantly lower than for those women who discontinue the drug. However, among patients who receive adjuvant tamoxifen therapy, individual responses are highly variable. These variable responses include the type, frequency and severity of endocrine symptoms and reduced tamoxifen adherence, which ultimately affect the cancer recurrence rate. Polymorphisms in some genes involved in the metabolism of tamoxifen are likely to influence these detrimental responses to tamoxifen.

**Aim of the study**

This study aims to characterize the profile of genetic polymorphisms of tamoxifen metabolism-associated genes in Chinese women with breast cancer and to explore the inter-relationships between genetic polymorphisms, endocrine symptoms, and adherence to tamoxifen that could affect therapeutic efficacy.

**Study plan**

The study consists of a 3 months feasibility study and a cohort study for 1 and half year.

230 Chinese breast cancer patients will be approached and recruited at the Department of Clinical Oncology in Prince of Wales Hospital for the study. 30 patients will be recruited in the feasibility study and 200 patients will be in the cohort study.

Inclusion criteria: Chinese women with histologically confirmed estrogen receptor-positive, stage I–III, primary invasive breast cancer treated with definitive surgery and chemotherapy and started on tamoxifen (20 mg daily) within the past month.

Exclusion criteria:

1. patients with other primary malignancies within the last 5 years,
2. patients who are pregnant or are planning on becoming pregnant, lactating,
3. treating with investigational drugs within the 4 weeks prior to enrolment,
4. those who are not able to give informed consent

A questionnaire consists of demographics, health status details, assessment of endocrine symptoms and drug adherence will be completed at enrolment. The participants will be assessed with the same questionnaire in 3, 6, 9 and 12, 15, and 18 months by phone interview. At 12 and 18 months, information on breast cancer recurrence will be collected.The saliva sample will also be collected from the participants at enrolment to test for drug metabolism enzymes and drug transporters. Participants will be required to keep a medication log book to record their intake of tamoxifen, supplements, Chinese herbal medicine, or other co-medication on a daily basis.

The surveys will be completed anonymously. There is no risk and benefit raised from the study. This Information Sheet with explanation of the study purpose and nature of participation together with the enclosed Consent Form is for your consideration. If you agree to participate, please sign the Consent Form.

Your participation in this study is highly appreciated and is entirely voluntary. You have every right to ask any questions and to decline participation at any time. You also have the right to refuse or withdraw from the study before or during the study. All information obtained will be kept confidentially and used for research purpose only. The research data will be stored safely and nobody can access the data without permission from the participants and the principal investigator. You have the right of access to the personal data and know study results when needed.

This study is organized by the Chinese University of Hong Kong and Prince of Wales Hospital. The research has been reviewed by The Joint Chinese University of Hong Kong-New Territories East Cluster Clinical Research Ethics Committee.

**Inquiry contact**

If you want to obtain further information about the research, please contact the principal investigator Prof Carmen Chan at 3943 6218

You will be given a copy of the information sheet and a signed copy of the informed consent form.
